# Inaugural Readmission Penalties for Total Hip and Total Knee Arthroplasty Procedures Under the Hospital Readmissions Reduction Program

**DOI:** 10.1001/jamanetworkopen.2019.16008

**Published:** 2019-11-22

**Authors:** Benjamin Y. Li, Kenneth L. Urish, Bruce L. Jacobs, Chang He, Tudor Borza, Yongmei Qin, Hye Sung Min, James M. Dupree, Chad Ellimoottil, Brent K. Hollenbeck, Mariel S. Lavieri, Jonathan E. Helm, Ted A. Skolarus

**Affiliations:** 1 Dow Division for Urologic Health Services Research, Department of Urology, University of Michigan, Ann Arbor; 2 Magee Bone and Joint Center, Department of Orthopaedic Surgery, University of Pittsburgh, Pittsburgh, Pennsylvania; 3 Department of Urology, University of Pittsburgh, Pittsburgh, Pennsylvania; 4 Michigan Society of Thoracic and Cardiovascular Surgeons Quality Collaborative, University of Michigan, Ann Arbor; 5 Department of Urology, University of Wisconsin, Madison; 6 Department of Industrial and Operations Engineering, University of Michigan, Ann Arbor; 7 Operations and Decision Technologies, Indiana University Kelley School of Business, Bloomington; 8 Health Services Research and Development, Center for Clinical Management Research, VA Ann Arbor Healthcare System, Ann Arbor, Michigan

## Abstract

**Importance:**

The Hospital Readmissions Reduction Program (HRRP) is a Centers for Medicare and Medicaid Services policy that levies hospital reimbursement penalties based on excess readmissions of patients with 4 medical conditions and 3 surgical procedures. A greater understanding of factors associated with the 3 surgical reimbursement penalties is needed for clinicians in surgical practice.

**Objective:**

To investigate the first year of HRRP readmission penalties applied to 2 surgical procedures—elective total hip arthroplasty (THA) and total knee arthroplasty (TKA)—in the context of hospital and patient characteristics.

**Design, Setting, and Participants:**

Fiscal year 2015 HRRP penalization data from Hospital Compare were linked with the American Hospital Association Annual Survey and with the Healthcare Cost and Utilization Project State Inpatient Database for hospitals in the state of Florida. By using a case-control framework, those hospitals were separated based on HRRP penalty severity, as measured with the HRRP THA and TKA excess readmission ratio, and compared according to orthopedic volume as well as hospital-level and patient-level characteristics. The first year of HRRP readmission penalties applied to surgery in Florida Medicare subsection (d) hospitals was examined, identifying 60 663 Medicare patients who underwent elective THA or TKA in 143 Florida hospitals. The data analysis was conducted from February 2016 to January 2017.

**Exposures:**

Annual hospital THA and TKA volume, other hospital-level characteristics, and patient factors used in HRRP risk adjustment.

**Main Outcomes and Measures:**

The HRRP penalties with HRRP excess readmission ratios were measured, and their association with annual THA and TKA volume, a common measure of surgical quality, was evaluated. The HRRP penalties for surgical care according to hospital and readmitted patient characteristics were then examined.

**Results:**

Among 143 Florida hospitals, 2991 of 60 663 Medicare patients (4.9%) who underwent THA or TKA were readmitted within 30 days. Annual hospital arthroplasty volume seemed to follow an inverse association with both unadjusted readmission rates (*r* = −0.16, *P* = .06) and HRRP risk-adjusted readmission penalties (*r* = −0.12, *P* = .14), but these associations were not statistically significant. Other hospital characteristics and readmitted patient characteristics were similar across HRRP orthopedic penalty severity.

**Conclusions and Relevance:**

This study’s findings suggest that higher-volume hospitals had less severe, but not significantly different, rates of readmission and HRRP penalties, without systematic differences across readmitted patients.

## Key Points

**Question:**

How are the inaugural penalties for surgical readmissions under the Hospital Readmissions Reduction Program of the Centers for Medicare and Medicaid Services associated with surgical volume and with hospital and patient characteristics?

**Findings:**

In this case-control study of 143 Florida hospitals, with 2991 readmitted Medicare patients, hospitals with a high volume of elective total hip and total knee arthroplasty procedures had lower, but not significantly different, readmission penalties than those with low volumes of these procedures. No other systematic differences were detected across hospitals or readmitted patients.

**Meaning:**

It seems that penalties for surgical readmissions under the Hospital Readmissions Reduction Program may be inversely associated with surgical volume, but this requires validation in a larger, nationwide cohort.

## Teaser

This case-control study evaluates the first year of penalties assessed by the Hospital Readmissions Reduction Program policy of the Centers for Medicare and Medicaid Services to investigate whether an association exists between penalties and surgical volume or characteristics of hospitals or patients undergoing elective total hip arthroplasty or total knee arthroplasty.

## Introduction

The Hospital Readmissions Reduction Program (HRRP) of the Centers for Medicare and Medicaid Services (CMS) began October 2012 in an effort to decrease readmissions within 30 days of hospitalization.^1,2,3^ As part of the Patient Protection and Affordable Care Act, the HRRP has evolved as a national health policy, progressively increasing its maximum penalty from 1% to 3% of total Medicare inpatient payments based on excess readmissions.^4,5,6^ Although the policy initially covered readmissions following 3 common medical conditions (acute myocardial infarction, heart failure, and pneumonia), the policy expanded in 2014 to include chronic obstructive pulmonary disease and its first surgical procedures: elective total hip arthroplasty (THA) and total knee arthroplasty (TKA).^1^ Given the hundreds of thousands of THA and TKA procedures performed each year, the implications of reducing readmission after these common orthopedic procedures are significant.^7,8,9^

However, concerns remain regarding the penalization method and spillover effects of the HRRP, especially as it expands to surgical, rather than solely medical, readmissions. For instance, safety-net and teaching hospitals are more likely to be penalized by the HRRP despite having better mortality outcomes.^10,11,12,13^ In addition, patient characteristics (eg, sociodemographic characteristics and performance status) not included in the case-mix adjustments of the policy may contribute to readmissions, leaving some hospitals unfairly penalized.^14^ For targeted medical conditions, changes in documentation standards may have inflated the reported association of the program with reducing readmission rates.^15^ This has spurred concern regarding similar dynamics in surgical procedures and future implementation of the HRRP.

The introduction of penalization may also exacerbate tensions at the hospital level between current practices and financial incentives. In 2013, through the Bundled Payments for Care Improvement Initiative, hospitals could choose to bundle payments for lower extremity joint replacement. Beginning in 2016, the Comprehensive Care for Joint Replacement model mandated bundled Medicare payments for THA and TKA from admission to 90 days after hospital discharge. These programs added further complexity at a time when hospitals began bearing the penalties for readmissions following HRRP-targeted surgical procedures.^16,17^ Since 2013, it is likely that hospitals prepared for the HRRP alongside those for bundled payment programs. Implementing surgical readmission penalties through the launch time of these quality improvement programs in orthopedic surgery created uncertainty at the hospital level and, to date, has not been well characterized.

For these reasons, the present study investigated whether HRRP penalties were associated with recognizable hospital and patient characteristics that might systematically disadvantage participating hospitals. We specifically examined readmission rates and HRRP penalties for elective THA and TKA procedures in the context of a common measure of orthopedic surgical quality: hospital arthroplasty volume.^18,19,20,21,22^ Since high arthroplasty volume has traditionally been associated with lower readmissions, our study provides a litmus test using real-world data for this recent readmissions policy. In this context, we hypothesized arthroplasty volume to be inversely associated with HRRP penalties for THA and TKA. Moreover, our investigation of patient-level characteristics also informs the completeness of the risk-adjustment algorithm of the program, which may be reassuring to practicing clinicians. Better understanding the implications of the HRRP for orthopedic surgery provides critical insights into intended and unintended consequences of including other surgical procedures, such as cardiac surgery.

## Methods

### Data Sources

We used 3 data sources across policy, hospital, and patient levels to conduct this study. First, we used the CMS quality-of-care reporting database, Hospital Compare, to identify Medicare subsection (d) hospitals participating in the HRRP in 2015, the inaugural year for THA and TKA penalties. Within each hospital, we focused on the excess readmission ratio (ERR) of the HRRP for THA and TKA. The ERR is a condition-specific metric, centered at 1.0. Because ERRs are calculated with lead-in patient data (ie, these measures are based on data from July 2010 to June 2013), we similarly examined the most recent 2-year (2012-2013) lead-in hospital and patient data for our study.^23^ Second, we linked CMS hospital identifier numbers and ERRs to corresponding American Hospital Association Annual Survey data to gather hospital characteristics. In our final hospital cohort, all hospitals reported their THA and TKA ERR in Hospital Compare data, and there was 98% overlap between hospitals from Hospital Compare and American Hospital Association data. Third, to study patient-level characteristics relevant to readmissions after THA and TKA, we merged CMS penalization data from Hospital Compare with the State Inpatient Database (SID) for Florida from the Healthcare Cost and Utilization Project. During our study period, Florida had the second-highest volume of THA and TKA of any state, accounting for 7% of all such procedures in the United States (eFigure 1 in the Supplement).^7,8,9^ Specifically, we used SID data to create a hospital-level summary of patient characteristics from 2012 to 2013.^24^ We used inclusion and exclusion criteria based on the HRRP method to define an elective THA and TKA patient cohort in the SID data.^23^ Collectively, these 3 data sources provided unique data to examine not only volume-outcomes associations for readmission after THA and TKA but also hospital and patient characteristics according to corresponding HRRP penalization data. This study followed the Strengthening the Reporting of Observational Studies in Epidemiology (STROBE) reporting guideline for case-control studies. Consistent with the policies of the University of Michigan for studies using deidentified administrative databases, the present study was deemed excluded from formal institutional review board evaluation, and the requirement for informed participant consent was waived.

### Hospital THA and TKA Volume

From Hospital Compare data, we identified and categorized 143 Florida hospitals into the following quartiles based on their (summed) elective THA and TKA volume: quartile 1 (25-114 discharges [n = 36]), quartile 2 (118-260 discharges [n = 36]), quartile 3 (269-592 discharges [n = 35]), and quartile 4 (595-2869 discharges [n = 36]). Hospitals performing less than 25 cases during the HRRP performance period were excluded from the HRRP and were similarly not included in this study.^23^

### Outcome Variables

Our primary outcome was the correlation between facility arthroplasty volume and THA and TKA ERR. This ERR was based on Medicare THA and TKA procedures performed by the hospital and contributed to the overall HRRP financial penalty of the hospital. Using Hospital Compare data for Florida, we identified the elective THA and TKA ERR of each hospital and created 4 categories of hospital penalty severity. We categorized hospitals as “no penalty” if their THA and TKA ERR was 1.000 or less (n = 67). That is, the THA and TKA risk-adjusted readmission rates of such hospitals were lower than expected; thus, there was no contribution to the overall HRRP penalty. We then stratified the remaining hospitals based on their potential for penalization into 3 categories: low penalty (1.000<ERR<1.059; n = 25), moderate penalty (1.059≤ERR<1.139; n = 25), or high penalty (ERR≥1.139; n = 26). In this way, the study followed a case-control research framework, with each case group corresponding to a different level of THA and TKA contribution to readmission penalty.

We next determined the extent to which hospital features described in the American Hospital Association data varied in association with our penalty severity categories. This analysis enabled us to determine whether systematic differences existed between penalized and nonpenalized hospitals. Building on previous work, we defined teaching hospitals as those with membership in the Council of Teaching Hospitals of the Association of American Medical Colleges, a residency training program approved by the Accreditation Council for Graduate Medical Education, or a ratio of full-time equivalent interns and residents to hospital beds of at least 0.25.^25,26^ We then obtained patient-level data and unadjusted 30-day readmission rates from the Florida SID. Because the HRRP uses patient case-mix variables to adjust readmission rates before calculating penalties, we used the SID data to investigate whether readmitted patient characteristics specifically used by HRRP risk adjustment varied in association with penalization. These case-mix risk variables included age, sex, procedure type (THA or TKA), congenital deformities of the hip (*International Classification of Disease, Ninth Revision, Clinical Modification* [*ICD-9-CM*] code 755.63), posttraumatic osteoarthritis (*ICD-9-CM* codes 716.15 and 716.16), and morbid obesity (*ICD-9-CM* code 278.01).^23^ We also included other characteristics potentially associated with THA and TKA readmission penalty severity, including the Charlson Comorbidity Index, race/ethnicity, index hospitalization length of stay, and readmission hospitalization length of stay.

For a sensitivity analysis, we examined whether unadjusted hospital readmission rates in SID data were associated with risk-adjusted hospital readmission rates according to the HRRP Hospital Compare database. These risk-adjusted readmission rates, termed *predicted readmission rates* by the HRRP, are a key factor in determining HRRP financial penalties. The purpose of this step was to examine whether or not the unadjusted readmission rate of a hospital was associated with its HRRP predicted readmission rate, potentially distributing penalties across the spectrum of unadjusted readmission rates and alleviating concerns about the HRRP risk-standardization, especially among hospitals with higher unadjusted readmission rates. For example, could a hospital unadjusted readmission rate of 6.1% be adjusted to a predicted readmission rate of 4.3%? Finally, because the aggregated HRRP financial penalty depends on ERRs from all HRRP-applicable medical conditions and surgical procedures, we compared the unadjusted orthopedic readmission rate of each hospital with its aggregated HRRP financial penalty to better understand the downstream ramifications of the HRRP risk-adjustment method. The HRRP penalizes the Medicare base operating diagnosis-related group payment amount, and the penalty is capped at 3%.^1^

### Statistical Analysis

In bivariate analyses, such as between facility orthopedic volume and either readmission rate or THA and TKA ERR, we calculated the Pearson correlation coefficient and tested it against the absence of association (*r* = 0). When investigating how hospital and patient characteristics were associated with THA and TKA penalty severity, we performed Pearson χ^2^ significance tests for categorical variables and Wilcoxon rank sum tests for continuous variables. For both hospital and patient characteristics, we excluded any missing values from the analysis and reported their frequencies. The probability of a type I error was set at .05, and all testing was 2-sided. We performed all analyses from February 2016 to January 2017 using SAS software, version 9.4 (SAS Institute Inc).

## Results

### ERR and Arthroplasty Volume

We identified 60 663 Medicare patients who underwent elective THA and TKA across 143 hospitals in Florida during 2012 and 2013, the 2 most recent lead-in years used to calculate the 2015 HRRP penalties. We found that the Medicare unadjusted 30-day arthroplasty readmission rate in Florida hospitals was 4.9%, accounting for 2991 patients. We report results for this readmitted Medicare patient cohort unless specifically noted (see eFigure 2 in the Supplement for patient inclusion/exclusion flowchart). Of the 143 hospitals in our study, 76 (53.1%) had readmission rates higher than expected for elective THA and TKA (ie, elective THA and TKA ERR > 1.000). All hospitals with excess THA and TKA readmissions incurred an aggregated, downstream financial penalty under the HRRP.

**Figure 1.**
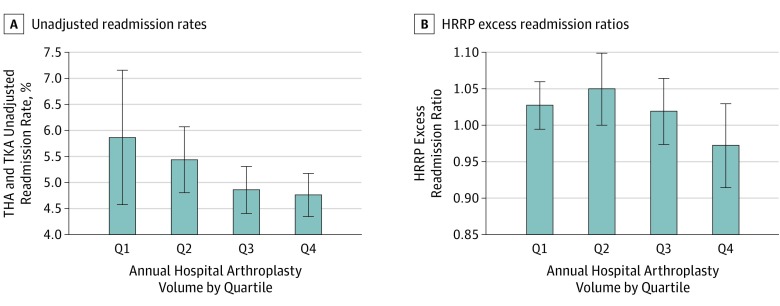
Unadjusted Readmission Rates and Hospital Readmissions Reduction Program (HRRP) Excess Readmission Ratios Associated With Hospital Arthroplasty Volume Elective total hip arthroplasty (THA) and total knee arthroplasty (TKA) unadjusted readmission rates were inversely associated with arthroplasty volume (*r* = −0.16, *P* = .06), as were HRRP risk-standardized readmission rates (*r* = −0.12, *P* = .14), but these associations were not statistically significant in our cohort. Q indicates quartile with Q1 being the lowest arthroplasty volume, and Q4 being the highest. Error bars indicate 95% CIs.

The median hospital THA and TKA volume was 260, ranging from 25 to 2869. Overall, the unadjusted readmission rate (*r* = −0.16, *P* = .06) and the ERR (*r* = −0.12, *P* = .14) were inversely associated with arthroplasty volume, but this association was not statistically significiant. As shown in Figure 1, the highest volume quartile was particularly protective for readmissions and HRRP penalties. Although the HRRP method excludes hospitals with fewer than 25 elective THA and TKA cases, we also found that hospitals just above this threshold (≤50 discharges) had relatively volatile unadjusted readmission rates, ranging from 0% to 21.2%.

### Hospital and Patient Characteristics Associated With HRRP Penalty Categories

We investigated whether HRRP ERRs were associated with hospital-level characteristics previously associated with quality of care.^27^ We found that hospitals did not vary significantly in association with elective THA and TKA penalty categories when compared with characteristics such as teaching hospital status, nurse to bed ratio, and proportion of Medicare or Medicaid facility days. However, as detailed in Table 1, the proportion of Medicaid facility days tended to be higher for hospitals in the moderate to high penalty categories. When considering Medicare and non-Medicare patient populations, both populations’ unadjusted orthopedic readmission rates were directly associated with orthopedic ERRs (Medicare: *r* = 0.44, *P* < .001; non-Medicare: *r* = 0.18, *P* = .04).

**Table 1.** Characteristics of 143 Hospitals by Elective THA and TKA ERRs in 2015

Characteristic	HRRP Elective THA and TKA Penalty, No. (%)^a^				*P* Value^b^
	None	Low	Moderate	High	
No. of hospitals^c^	67 (46.8)	25 (17.5)	25 (17.5)	26 (18.2)	
Hospital bed size					
Small (<400)	8 (11.9)	4 (16.0)	2 (8.0)	3 (11.5)	.56
Medium (400-599)	37 (55.3)	14 (56.0)	10 (40.0)	11 (42.3)	
Large (≥600)	22 (32.8)	7 (28.0)	13 (52.0)	12 (46.2)	
Total surgical operations by quartile					
1 (<4990)	17 (25.3)	8 (32.0)	4 (16.0)	6 (23.1)	.35
2 (4990-6636)	17 (25.4)	7 (28.0)	4 (16.0)	7 (26.9)	
3 (6637-10 198)	17 (25.4)	3 (12.0)	12 (48.0)	5 (19.2)	
4 (≥10 199)	16 (23.9)	7 (28.0)	5 (20.0)	8 (30.8)	
Nurse-to-bed ratio by quartile					
1 (<0.97)	18 (26.9)	7 (28.0)	5 (20.0)	6 (23.1)	.56
2 (0.97-1.18)	16 (23.9)	8 (32.0)	4 (16.0)	8 (30.7)	
3 (1.19-1.51)	13 (19.4)	7 (28.0)	8 (32.0)	8 (30.8)	
4 (≥1.52)	20 (29.8)	3 (12.0)	8 (32.0)	4 (15.4)	
Teaching hospital	13 (19.4)	4 (16.0)	5 (20.0)	6 (23.1)	.96
Metropolitan setting	46 (68.7)	16 (64.0)	16 (64.0)	18 (69.2)	.95
Private, for-profit ownership	28 (41.8)	11 (44.0)	14 (56.0)	17 (65.4)	.18
Medicare proportion of total facility days by quartile					
1 (≤0.46)	16 (23.9)	3 (12.0)	10 (40.0)	7 (26.9)	.61
2 (0.47-0.53)	17 (25.4)	6 (24.0)	6 (24.0)	7 (26.9)	
3 (0.54-0.63)	19 (28.3)	8 (32.0)	3 (12.0)	6 (23.1)	
4 (>0.63)	15 (22.4)	8 (32.0)	6 (24.0)	6 (23.1)	
Medicaid proportion of total facility days by quartile					
1 (≤0.12)	14 (20.9)	11 (44.0)	6 (24.0)	5 (19.2)	.07
2 (0.13-0.19)	21 (31.3)	6 (24.0)	4 (16.0)	4 (15.4)	
3 (0.20-0.22)	20 (29.9)	5 (20.0)	5 (20.0)	7 (26.9)	
4 (>0.22)	12 (17.9)	3 (12.0)	10 (40.0)	10 (38.5)	

Abbreviations: ERR, excess readmission ratio; HRRP, Hospital Readmissions Reduction Program; THA, total hip arthroplasty; TKA, total knee arthroplasty.

^a^ No penalty, ERR ≤ 1.000; low penalty, 1.000 < ERR < 1.059; moderate penalty, 1.059 ≤ ERR < 1.139; high penalty, ERR ≥ 1.139.

^b^ For χ^2^ test or Wilcoxon rank sum test.

^c^ Presented as frequencies with row percentage.

Patients readmitted following THA and TKA in Florida hospitals were similar with regard to age, sex, race/ethnicity, household income, and index hospitalization length of stay across our HRRP penalty categories. Among readmitted patients, we found no differences in patient-level risk variables used by the HRRP method across penalty groups (Table 2).

**Table 2.** Characteristics of 2991 Readmitted Patients by Elective THA and TKA ERR in 2015

Characteristic^a^	HRRP Elective THA and TKA Penalty, No. (%)^b^				*P* Value^c^
	None	Low	Moderate	High	
Age, mean (SE), y	73.6 (0.27)	73.9 (0.37)	74.0 (0.33)	73.3 (0.35)	.69
Women	683 (58.1)	305 (59.0)	321 (54.9)	424 (59.5)	.52
TKA	758 (64.5)	350 (67.7)	411 (70.3)	452 (63.4)	.57
Morbid obesity^d^	65 (5.5)	39 (7.5)	27 (4.6)	45 (6.3)	.63
Charlson Comorbidity Index^e^					
0	887 (75.5)	382 (73.9)	445 (76.0)	503 (70.5)	.31
1	192 (16.3)	92 (17.8)	96 (16.4)	131 (18.4)	
2	73 (6.2)	34 (6.6)	32 (5.5)	54 (7.6)	
≥3	24 (2.0)	9 (1.7)	12 (2.1)	25 (3.5)	
Race/ethnicity					
White	1003 (85.2)	442 (85.3)	446 (76.1)	549 (77.0)	.28
Black	68 (5.8)	40 (7.8)	38 (6.5)	69 (9.7)	
Hispanic	83 (7.1)	20 (3.9)	86 (14.8)	78 (11.0)	
Asian	2 (0.2)	2 (0.4)	5 (0.9)	6 (0.8)	
Native American	2 (0.2)	0	1 (0.2)	0	
Other	14 (1.2)	8 (1.6)	6 (1.0)	4 (1.0)	
Missing	4 (0.3)	5 (1.0)	3 (0.5)	7 (1.0)	
Median annual household income					
Quartile 1 (low)	375 (31.9)	164 (31.7)	192 (32.9)	233 (32.6)	.82
Quartile 2	447 (38.0)	158 (30.6)	157 (26.8)	218 (30.6)	
Quartile 3	251 (21.3)	133 (25.7)	158 (27.0)	190 (26.7)	
Quartile 4 (high)	89 (7.6)	52 (10.1)	68 (11.6)	61 (8.6)	
Missing	14 (1.2)	10 (1.9)	10 (1.7)	11 (1.5)	
Hospitalization length of stay, mean (SE), d					
Index	3.76 (0.06)	3.92 (0.11)	3.95 (0.11)	3.92 (0.09)	.27
Readmission	4.31 (0.09)	4.44 (0.14)	4.44 (0.13)	4.23 (0.11)	.58

Abbreviations: ERR, excess readmission ratio; HRRP, Hospital Readmissions Reduction Program; THA, total hip arthroplasty; TKA, total knee arthroplasty.

^a^ Few patients in our study underwent 2 simultaneous arthroplasty procedures, had other congenital deformity of the hip, or had posttraumatic osteoarthritis. Because these risk variables were very rare in patients receiving elective THA and TKA, they are not reported in this table in accordance with Healthcare Cost and Utilization Project rules.

^b^ No penalty, ERR ≤ 1.000; low penalty, 1.000 < ERR < 1.059; moderate penalty, 1.059 ≤ ERR < 1.139; high penalty, ERR ≥ 1.139.

^c^ For χ^2^ or Wilcoxon rank sum test.

^d^ Indicated by *International Classification of Disease, Ninth Revision, Clinical Modification* code 278.01.

^e^ The lower the index, the lower the risk of mortality and the better the patient’s prognosis.

**Figure 2.**
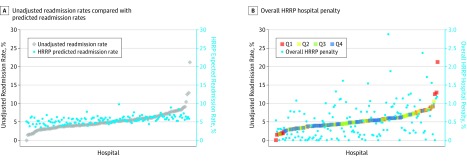
Unadjusted Readmission Rates Compared With Predicted Readmission Rates and Overall Hospital Readmissions Reduction Program (HRRP) Hospital Penalty in 143 Hospitals The HRRP predicted readmission rates for elective total hip arthroplasty (THA) and total knee arthroplasty (TKA), key parameters in calculating excess readmission ratios, trend with unadjusted elective THA and TKA readmission rates (*r* = 0.41, *P* < .001) but have less variance (1.00 vs 5.82; *P* < .001). Overall hospital penalties administered by the HRRP are weighted toward hospitals with higher unadjusted elective THA and TKA readmission rates (*r* = 0.38, *P* < .001). Low volume (≤50 discharges) arthroplasty centers have relatively volatile unadjusted readmission rates (range, 0%-21.2%). Q indicates quartile with Q1 being the lowest arthroplasty volume, and Q4 being the highest.

In our sensitivity analyses, risk-adjusted readmission rates from CMS models and from Hospital Compare trended with the hospital unadjusted readmission rates from the SID data (*r* = 0.41, *P* < .001). Of the 143 hospitals, 49 (34.3%) had a risk-adjusted readmission rate less than their unadjusted readmission rate. This indicated that approximately one-third of hospitals experienced downward patient-level adjustments to their unadjusted readmission rate (range of readmission rate adjustments, −15.41 to 5.00) (Figure 2A). As illustrated in Figure 2B, we found that the aggregated hospital penalties administered by the HRRP were weighted toward hospitals with higher unadjusted orthopedic readmission rates (*r* = 0.38, *P* < .001). Furthermore, hospitals in the upper half of unadjusted readmission rates received nearly twice the mean aggregated financial penalty as hospitals in the lower half of unadjusted readmission rates (0.37% vs 0.67%; *P* < .001).

## Discussion

Since 2012, the HRRP has grown as a national readmissions health policy lever. In 2014, the HRRP began including readmissions following elective THA and TKA as its first application to surgery. Two years later, the program broadened its scope to include coronary artery bypass grafting. Our study found that high-volume arthroplasty centers had lower, but not significantly different, unadjusted readmission rates and ERRs than low-volume centers. We detected no differences in hospital-level and readmitted patient-level characteristics across HRRP penalty categories, including proportion of Medicare days, teaching hospital status, and measures of patient comorbidity. This finding suggests that factors contributing to HRRP penalties, other than surgical volume, are not routinely captured in survey and administrative data. These factors can have implications, particularly on understanding the effect of health policies on the hospital, department, and surgeon level. Better understanding of the complex contextual factors contributing to readmissions after surgery appears warranted to improve performance.

Our results showed that many reportable hospital-level features did not trend with THA and TKA ERRs, and this is consistent with the CMS decision to not adjust for specific hospital characteristics in determining orthopedic surgery readmission penalties. It is possible that organizational factors that underlie readmission quality of care cannot be fully captured by the variables included in our study, although we included a variety of commonly studied characteristics relevant to readmission.^28^ An interesting line for future research involves aggregating outcomes data and hospital characteristics on a county level. This would provide more encompassing insights into how factors such as hospital density and Medicaid participation rate are associated with surgical readmission penalties.

The present study examined readmitted patient-level factors with respect to surgical ERR magnitudes. The patient case-mix variables that we studied, including comorbidities and obesity, were not associated with HRRP penalty categories. The HRRP method was designed to adjust for patient case mix when calculating THA and TKA ERRs; thus, the lack of associations between ERRs and patient case-mix variables is intuitive. In addition, previous investigations have reported overpenalization of safety-net hospitals based on excess medical readmissions and sicker patients.^10,11,12,13^ This has spurred debate about whether the HRRP should adjust for socioeconomic status given the possible association with excess readmissions.^29,30^ We did not study safety-net hospitals directly, but we found surgical ERRs did not trend with median household income, a heuristic for socioeconomic status.

Since the HRRP has taken effect, there has been an accelerated decrease in Medicare readmissions.^31,32,33^ Going forward, understanding and acting on the underlying factors associated with this decrease, for medical and surgical readmissions, are equally important. That being said, there is concern that the costs of thorough readmissions reduction interventions may be unsustainable^34^ and that too narrow a focus on reducing hospital readmissions may introduce externalities in the form of spillover effects or increased postdischarge use.^31,35^ One potential bulwark against negative consequences arises in the HRRP aggregation method: with each additional applicable condition having an ERR greater than 1.000, the hospital faces a larger financial penalty under the HRRP that year. Thus, as the HRRP expands, hospital leadership may be further incentivized to translate best practices across disparate teams and departments.

### Limitations

This investigation should be interpreted in the context of several limitations. First, we designed our study to mirror the HRRP method in using lead-in patient data to determine the inaugural year of HRRP penalties for surgical readmissions.^23^ The retrospective design and data derivation from administrative data sets limit the ability of our causal inference. However, merging 3 data sets across time enabled us to address questions that may be immediately relevant to hospitals and surgeons facing the policy (eg, our examination of the association between arthroplasty volume and penalties). Second, we used the SID to connect patient characteristics to readmission penalties. Although we followed the HRRP method as closely as possible, we acknowledge that there are inconsistencies between the Medicare patient cohort used in the HRRP model and the SID patient cohort used in the present study. Because our findings regarding unadjusted readmission rates are consistent with previously reported readmission rates after THA and TKA, the SID patient cohort is likely a close intersection with the CMS cohort.^36,37,38,39^ In addition, using an all-payer database enabled us to examine the potential for spillover effects from Medicare to non-Medicare populations. Third, this investigation focused on the first year of HRRP penalties for 2 surgical procedures in Florida, limiting the power of our inference. Further investigation may elucidate how these results generalize to other applicable conditions and readmission penalty contexts of the HRRP elsewhere in the United States. For example, other researchers have found similar volume-outcomes associations for orthopedic surgery in New York State.^40^ However, single-state studies may be limited by their sample size, and leveraging the Nationwide Readmissions Database may provide a richer picture of geographical variation in readmission penalties as well as further characterize the association between penalties and patient-level factors. Because we investigated the inaugural year of HRRP penalties for surgical readmissions, our findings may serve as a baseline for comparison as the implications of this policy evolve for hospitals performing major orthopedic and cardiac surgery. For example, the hospital response to readmission penalties following orthopedic surgery may shift with the addition of more surgical procedures or with the implementation of bundled payment programs, such as the Comprehensive Care for Joint Replacement model.

## Conclusions

We believe that our study helps connect hospital and patient characteristics to the first application of the HRRP to surgical procedures. We found that high-volume arthroplasty centers fared relatively better than low-volume centers and that neither patient-level nor hospital-level factors were associated with the adjusted readmission ratios used by the HRRP to administer penalties. Taken together, our findings related traditional measures (eg, facility arthroplasty volume, hospital- and patient-level characteristics) with newer, nationally standardized approaches to measure quality of care (eg, THA and TKA ERR). These findings provide additional context for clinicians, hospitals, and policy makers. A better understanding of the root factors associated with these observations for HRRP surgical procedures, and whether they are associated with other high-volume surgical procedures, (ie, cardiac) or payment policies (such as bundled reimbursements) appears to be warranted.

## Notes

Supplement. **eFigure 1.** Top 20 States by Estimated Medicare THA & TKA Volume Across the United States in 2012 **eFigure 2.** Patient Inclusion/Exclusion Flowchart
