# Supplementary material for: Inaugural Readmission Penalties for Total Hip and Total Knee Arthroplasty Procedures Under the Hospital Readmissions Reduction Program
Source: JAMA Netw Open. 2019 Nov 22;2(11):e1916008. doi: 10.1001/jamanetworkopen.2019.16008 (PMC6902819; doi:10.1001/jamanetworkopen.2019.16008)
Supplement: Supplement. — eFigure 1. Top 20 States by Estimated Medicare THA & TKA Volume Across the United States in 2012 eFigure 2. Patient Inclusion/Exclusion Flowchart [file jamanetwopen-2-e1916008-s001.pdf]

Supplementary Online Content

Li BY, Urish KL, Jacobs BL, et al. Inaugural readmission penalties for total hip and total knee arthroplasty procedures under the Hospital Readmissions Reduction Program. *JAMA Netw Open*. 2019;2(11):e1916008. doi:10.1001/jamanetworkopen.2019.16008

eFigure 1. Top 20 States by Estimated Medicare THA & TKA Volume Across the United States in 2012

eFigure 2. Patient Inclusion/Exclusion Flowchart

This supplementary material has been provided by the authors to give readers additional information about their work.

© 2019 Li BY et al. *JAMA Network Open*.

eFigure 1. Top 20 States by Estimated Medicare THA & TKA Volume Across the United States in 2012

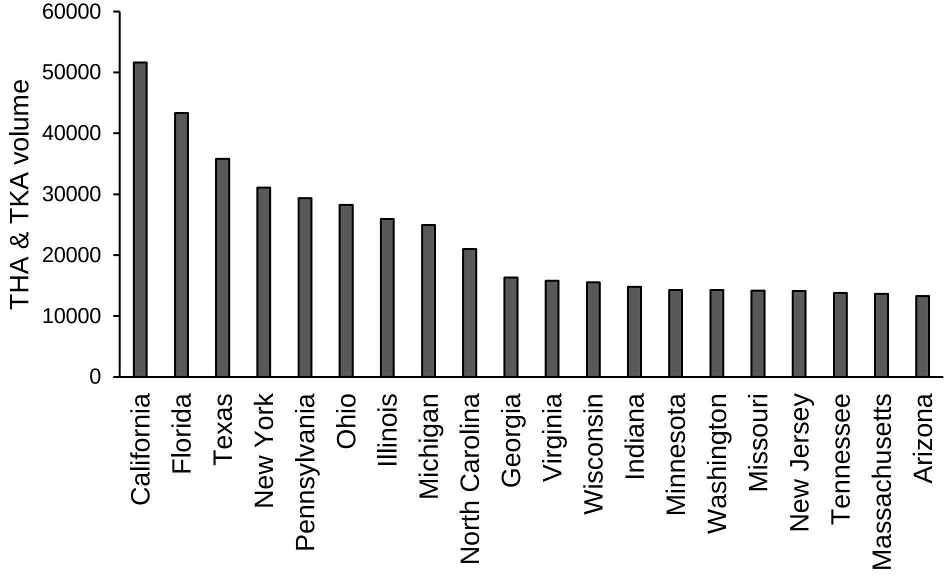

Abbreviations: THA & TKA; total hip arthroplasty & total knee arthroplasty

Data estimated by state from the Henry J. Kaiser Family Foundation total number of Medicare beneficiaries and Dartmouth Atlas of Health Care number of THA & TKA operations in 2012.[7-9]

© 2019 Li BY et al. *JAMA Network Open*.

eFigure 2. Patient Inclusion/Exclusion Flowchart

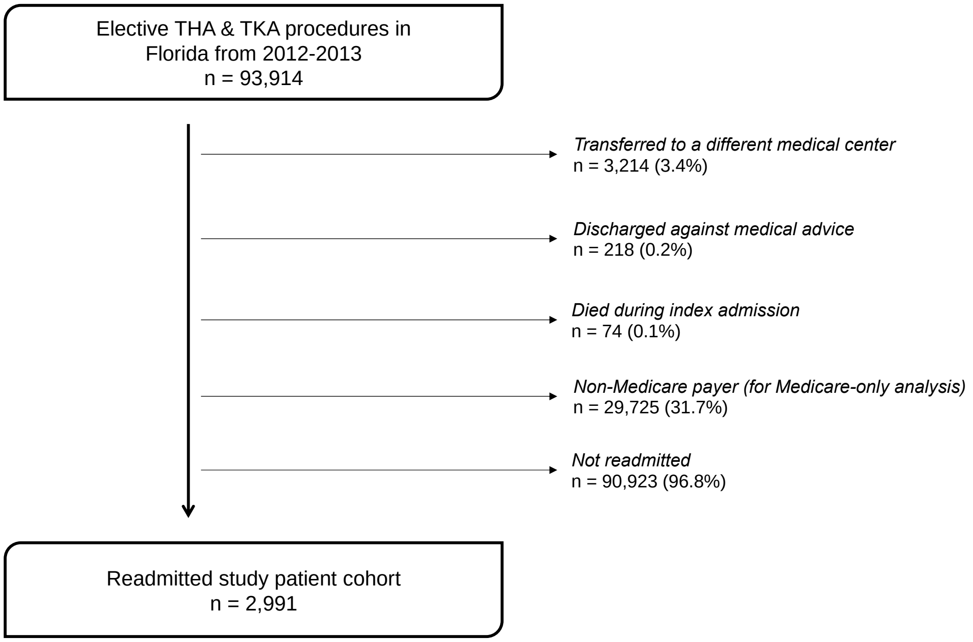

© 2019 Li BY et al. *JAMA Network Open*.
